# Das Rezidivmuster nach perkutaner Strahlentherapie mit und ohne zusätzliche fokale Dosiseskalation innerhalb der randomisierten FLAME-Studie für das lokale Prostatakarzinom

**DOI:** 10.1007/s00066-022-01955-w

**Published:** 2022-05-17

**Authors:** Simon K. B. Spohn, Anca-Ligia Grosu

**Affiliations:** Klinik für Strahlenheilkunde, Universitätsklinikum Freiburg, Robert-Koch-Str. 3, 79106 Freiburg, Deutschland

## Hintergrund

Die Strahlentherapie (RT) bietet Patienten mit lokalisiertem Prostatakarzinom aller Risikostadien (PCa) eine effektive und sichere kurative Behandlungsoption. Da eine homogene Erhöhung der Strahlendosis durch die Belastung der umgebenden Risikoorgane („organs at risk“ [OAR]) limitiert ist, bietet die fokale Dosiseskalation (FDE) auf die intraprostatische Tumormasse (GTV) eine interessante Möglichkeit, die lokale Tumorkontrolle zu erhöhen und gleichzeitig die Dosisvorgaben für die OAR zu respektieren. Studien, die einen Vergleich zwischen pathologischen Großflächenschnitten und Schnittbildgebung durchführten, haben gezeigt, dass die multiparametrische Magnetresonanztomographie (mpMRT) in der Lage ist, PCa-Läsionen mit ausreichender Sensitivität und Spezifität darzustellen, und somit eine Voraussetzung der GTV-Definition zur fokalen Dosiseskalation bietet.

Die Studie Focal Lesion Ablative Microboost in Prostate Cancer (FLAME) hat dieses Konzept in einer randomisierten, kontrollierten Phase-III-Studie untersucht und Patienten mit lokalisiertem, intermediärem oder Hochrisiko-PCa entweder mit 77 Gy in 35 Fraktionen oder mit zusätzlichem fokalem Boost (auf das GTV) bis zu 95 Gy in 35 Fraktionen behandelt [1]. Das GTV wurde anhand der T2-diffusionsgewichteten und kontrastmittelverstärkten Sequenzen der mpMRT definiert. Die Einhaltung der Dosisvorgaben für die OAR wurde priorisiert, sodass die Dosiseskalation reduziert werden konnte, falls erforderlich. Die Publikation der 5‑Jahres-Ergebnisse in 2021 hat eine signifikante Verbesserung des biochemisch rezidivfreien Überlebens (bRFS) ohne signifikante Erhöhung der Toxizität durch die fokale Dosiseskalation ergeben.

Aufgrund der Tumorbiologie des PCa mit vergleichsweise langsamem Krankheitsverlauf hat sich zur Evaluation des Gesamtüberlebens (OS) nach Behandlung der validierte Surrogatendpunkt metastasenfreies Überleben (MFS) etabliert. Patienten mit Hochrisiko-PCa erleiden häufiger distante Metastasen, sodass sich die Frage stellt, ob dies auf eine bereits bei initialer Behandlung bestehende Mikrometastasierung oder auf eine unzureichende lokale Tumorkontrolle zurückzuführen ist. Aus diesem Grund wurde in der hier kommentierten FLAME-Studie der Effekt der fokalen Dosiseskalation auf die lokale Kontrolle („local failure-free survival“ [LFS]) und die regionale und distante Kontrolle („regional + distant metastasis-free survival“ [rdMFS]) untersucht.

## Patienten und Methoden

Zwischen 2009 und 2015 wurden Patienten mit intermediärem oder Hochrisiko-PCa in drei Zentren der Niederlande (Amsterdam und Nijmegen) und Belgiens (Leuven) eingeschlossen. Ausschlusskriterien waren u. a. Hinweise von Lymphknoten- oder Fernmetastasen, basierend auf konventionellem Staging, eine transurethrale Prostataresektion innerhalb von 3 Monaten vor der Strahlentherapie, ein International Prostate Symptom Score (IPSS) ≥ 20 Punkte und eine Kontraindikation für eine mpMRT oder nicht sichtbare GTV in der mpMRT. Alle Patienten erhielten Goldmarker zur Positionierungskontrolle während der bildgeführten Strahlentherapie (IGRT). Eine elektive Bestrahlung der pelvinen Lymphabflusswege wurde nicht vorgenommen. Das bRFS wurde anhand der Phoenix-Kriterien bestimmt. Da sich die klinische Praxis während der Durchführung der Studie – insbesondere durch die Etablierung der Positronenemissionstomographie (PET) mit Tracern gegen das prostataspezifische Membranantigen (PSMA) – verändert hatte, hing die Durchführung der Bildgebung von den behandelnden Ärzten und den jeweiligen Zentren ab.

Das Rezidivmuster wurde anhand der verwendeten bildgebenden Methoden während der Studiennachverfolgung mit deskriptiver Statistik analysiert. Es wurde zwischen Lokalrezidiven („local failure“ [LF]), regionalen Rezidiven („regional metastatic failure“ [rMF], definiert als regionale Lymphknotenmetastasen ohne Lokalrezidive oder Fernmetastasen) und distanten Rezidiven („distant metastatic failure“ [dMF], definiert als distante Metastasen ohne regionale Lymphknotenmetastasen oder Lokalrezidive) unterschieden. Für die Überlebensanalysen wurden regionale und distante Metastasen zu rdMF zusammengefasst. Unterschiede zwischen LFS und rdMFS wurden mittels Kaplan-Meier-Analysen und „log-rank t‑test“ bestimmt. Unter Berücksichtigung klinischer und pathologischer Parameter (Gleason-Score, T‑Kategorie, initialer PSA-Wert und Hormontherapie) wurden Cox-Regressionen durchgeführt. Zudem erfolgten Korrelationen zwischen der D98 % des GTV und den Rezidivendpunkten, um eine Dosis-Wirkungs-Kurve zur Vorhersage der Wahrscheinlichkeit von LF und rdMF bis zu 7 Jahre nach RT erstellen zu können.

## Ergebnisse

571 Patienten, davon 84 % mit hohem und 16 % mit intermediärem Risiko nach EAU-Kriterien, wurden in die FLAME-Studie eingeschlossen. 65 % der Patienten erhielten eine Androgendeprivation (ADT). In die Per-Protokoll-Analyse wurden 271 Patienten im Kontrollarm und 264 im experimentellen Arm behandelt. Die Bildgebung bei den insgesamt 95 Rezidiven umfasste Knochenszintigraphie (11 %), Computertomographie (CT, 13 %), Cholin-PET (13 %), PSMA-PET (72 %) sowie die mpMRT (26 %).

Im Vergleich zum Standardarm traten im experimentellen Arm seltener Lokalrezidive (21 vs. 7), regionale Rezidive (22 vs. 7) und distante Rezidive auf (34 vs. 25, davon distante Lymphknotenmetastasen 13 vs. 11, Knochenmetastasen 15 vs. 12 und viszerale Metastasen 6 vs. 2). Im experimentellen Arm wurden seltener Therapien wegen eines Rezidivs durchgeführt (26 vs. 40). LFS (*p* = 0,008) und rdMFS (*p* = 0,02) waren signifikant unterschiedlich in beiden Armen. Die Cox-Regression zeigte einen Vorteil für die fokale Dosiseskalation mit einer adjustierten Hazard Ratio (HR) von 0,33 (95 %-Konfidenzintervall [CI] 0,14–0,80) für LFS und 0,56 (95 %-CI 0,34–0,91) für rdMFS. Die Dosis-Wirkungs-Kurven zeigten eine geringere Vorhersagekraft für die Entstehung von LF und rdMF mit steigender Dosis, welche bei fokalen Boost-Dosen ≥ 85 Gy 0 % für LF und < 10 % für rdMF gegenüber 15 % nach Standarddosierung von 77 Gy betrug.

## Schlussfolgerung der Autoren

Eine fokale Dosiseskalation der mpMRT-definierten GTV bis zu 95 Gy verbessert das LFS und rdMFS signifikant und deutlich. Eine Behandlung des Primärtumors mit höheren Dosen reduziert das Risiko für die Entstehung von regionalen und distanten Metastasen. Diese Ergebnisse unterstützen die Hypothese, dass eine unzureichende Behandlung des Primärtumors zur Entstehung von Rezidiven beiträgt. Zudem hat eine Dosiseskalation auch unterhalb von 95 Gy positive Auswirkungen auf das LFS und rdMFS. Die verbleibende Rate von rdMF könnte möglicherweise auf bereits initial bestehende Mikrometastasen zurückzuführen sein.

## Kommentar

Die FLAME-Studie hat mit ihren beeindruckenden Ergebnissen gezeigt, dass eine fokale Dosiseskalation die Ergebnisse nach primär definitiver Strahlentherapie von intermediären und Hochrisiko-PCa-Patienten weiter verbessert. Die hier vorliegende Studie unterstreicht, dass eine fokale Dosiseskalation als neuer Therapiestandard in der EBRT akzeptiert werden sollte. Es ist davon auszugehen, dass sich die positiven Effekte der fokalen Dosiseskalation auch auf das Gesamtüberleben übertragen werden.

Erfreulicherweise scheint der fokale Boost keine signifikanten Auswirkungen auf das Toxizitätsprofil zu haben [1]. Zudem konnte das FLAME-Konsortium in zwei kürzlich veröffentlichten Analysen eine Dosis-Wirkungs-Beziehung für das Auftreten von urogenitaler und rektaler Toxizität und die applizierte Dosis auf Blase, Urethra und Rektum zeigen [2, 3]. Folglich kann durch Einhaltung strengerer Dosisvorgaben möglicherweise die Entstehung von Nebenwirkungen weiter reduziert und damit eine verbesserte Lebensqualität gewährleistet werden. In diesem Kontext sollte der Effekt einer fokalen Dosiseskalation gegenüber möglicher Toxizität abgewogen werden.

Die Autoren dieser Studie konnten zum ersten Mal zeigen, dass sich eine fokale Dosiseskalation positiv auf die Entstehung regionaler und distanter Metastasen auswirkt. Bei der Interpretation dieser Ergebnisse muss berücksichtigt werden, dass der hier analysierte Endpunkt rdMFS nicht mit dem als OS-Surrogatparameter validierten Endpunkt MFS gleichzusetzten ist. Dieser wurde von der Initiative *Intermediate Clinical Endpoint in Carcinoma of the Prostate* als ausschließliches Auftreten von distanten Metastasen definiert und basiert auf konventioneller Bildgebung. Da die die Implementierung der PSMA-PET in die Rezidivdiagnostik zu einer früheren und vermehrten Detektion regionaler und distanter Metastasen führt, müssen zukünftige Studien die Bedeutung von regionalem und distantem metastasenfreiem Überleben in der PSMA-PET-Ära analysieren. Erwähnenswert ist, dass die Autoren eine Dosis-Wirkungs-Beziehung für die Reduktion regionaler und distanter Metastasen finden und beschreiben konnten, sodass Patienten auch von einem Boost mit reduzierten Dosen (≥ 85 Gy) zu profitieren scheinen. Die niedrigen Raten an regionalen Rezidiven im fokalen Dosiseskalationsarm stellen zudem den „benefit“ einer zusätzlichen elektiven Bestrahlung der pelvinen Lymphabflusswege [4] und die Intensivierung einer konkomitanten Systemtherapie [5] infrage, da in der FLAME-Studie keine pelvine Bestrahlung durchgeführt wurde und nur relativ wenige Patienten eine ADT erhielten.

Während die insgesamt beeindruckenden Ergebnisse die Grundlage für eine schon heute sehr gute Therapie für Patienten mit primärem lokalisiertem Hochrisiko-PCa bieten, zeigt sie erneut, dass zusätzliche prädiktive Marker für eine verbesserte Risikostratifizierung notwendig sind, um eine optimale und personalisierte Therapie zu finden. Eine Möglichkeit hierzu bietet die heutzutage auch in der Primärdiagnostik von Hochrisikopatienten als Standard anerkannte PSMA-PET/CT [6], die durch ihre verbesserte Detektion nodaler und distanter Metastasen das primäre Therapiemanagement maßgeblich beeinflusst.

Zusätzlich ermöglicht die PSMA-PET ein verbessertes intraprostatisches Staging. Aufgrund der höheren Sensitivität in der Detektion der intraprostatischen Tumormasse und der weniger anspruchsvollen GTV-Definition im Vergleich zur mpMRT bietet die Implementierung der PSMA-PET zur fokalen Dosiseskalation die Möglichkeit einer besseren Tumorabdeckung und damit möglicherweise eine weitere Verbesserung der Rate lokaler, regionaler und distanter Rezidive [7]. Ziel zukünftiger Forschung muss die Extraktion von radiomischen Merkmalen und die Analyse dieser und etablierter Merkmale auf Basis von künstlicher Intelligenz sein, um die nichtinvasive Charakterisierung des PCa zu verbessern und mit der Entwicklung prognostischer und prädiktiver Marker eine verbesserte Risikostratifizierung und Patientenselektion zu ermöglichen [8].

Schließlich haben sich in den vergangenen Jahren die moderat hypofraktionierten RT-Konzepte (MHRT) und die stereotaktische Strahlentherapie (SBRT) in der Behandlung des PCa etabliert. Während für Patienten mit niedrigem und intermediärem Risiko von einer Gleichwertigkeit dieser Therapieregime mit einer konventionell fraktionierten RT ausgegangen werden kann, ist dies bei Hochrisikopatienten noch nicht abschließend geklärt. Die Ergebnisse der englischen PACE-C(NCT01584258)-Studie werden zur Beantwortung dieser Frage beitragen. Ob die Behandlung von Hochrisikopatienten auch mit diesen modernen Therapieregimen der fokalen Dosiseskalation erreicht wird, wird u. a. die HypoFocal-SBRT-Studie untersuchen, in der die fokal dosiseskalierte SBRT auf Basis der Implementierung der PSMA-PET und mpMRT analysiert wird [9].

## Fazit

- Eine fokale Dosiseskalation in der EBRT verbessert nicht nur die Rate biochemischer Rezidive, sondern auch lokaler, regionaler + distanter Metastasen.
- Es besteht eine Dosis-Wirkungs-Beziehung zwischen fokaler Boost-Dosis und dem Auftreten von lokalen, regionalen und distanten Metastasen. Wir dürfen also davon ausgehen, dass auch Dosiseskalationen unterhalb von 95 Gy (≥ 85 Gy) mit hoher Wahrscheinlichkeit einen positiven Therapieeffekt haben.
- Weitere prädiktive Marker zur Verbesserung der Risikostratifizierung sind notwendig, um Patienten zu identifizieren, die in der Ära der PSMA-PET und fokalen Dosiseskalation von einer Therapieeskalation bzw. -deeskalation profitieren dürften.
- Die HypoFocal-SBRT-Studie wird die SBRT mit fokaler Dosiseskalation auf Basis der PSMA-PET und mpMRT bei intermediären und Hochrisiko-PCa-Patienten analysieren.

*Simon K.B. Spohn und Anca-Ligia Grosu, Freiburg/Brsg.*
